# Evaluation of β-Sitosterol Loaded PLGA and PEG-PLA Nanoparticles for Effective Treatment of Breast Cancer: Preparation, Physicochemical Characterization, and Antitumor Activity

**DOI:** 10.3390/pharmaceutics10040232

**Published:** 2018-11-15

**Authors:** Moses Andima, Gabriella Costabile, Lorenz Isert, Albert J. Ndakala, Solomon Derese, Olivia M. Merkel

**Affiliations:** 1 Department of Pharmacy, Pharmaceutical Technology & Biopharmaceutics, Ludwig-Maximilian University of Munich, Butenandtstr. 5-13, 81377 Munich, Germany; andima.moses@gmail.com (M.A.); gabriella.costabile@cup.uni-muenchen.de (G.C.); loisph@cup.uni-muenchen.de (L.I.); 2 Department of Chemistry, University of Nairobi, P.O. Box 30197-00100, Nairobi, Kenya; andakala@uonbi.ac.ke (A.J.N.); sderese@uonbi.ac.ke (S.D.); 3 Department of Chemistry, Busitema University, P.O. Box 236, Tororo, Uganda

**Keywords:** β-Sitosterol, PLGA nanoparticles, PEG-PLA nanoparticles, breast cancer

## Abstract

β-Sitosterol (β-Sit) is a dietary phytosterol with demonstrated anticancer activity against a panel of cancers, but its poor solubility in water limits its bioavailability and therapeutic efficacy. In this study, poly(lactide-co-glycolic acid) (PLGA) and block copolymers of poly(ethylene glycol)-block-poly(lactic acid) (PEG-PLA) were used to encapsulate β-Sit into nanoparticles with the aim of enhancing its in vitro anticancer activity. β-Sitosterol-loaded PLGA and PEG-PLA nanoparticles (β-Sit-PLGA and β-Sit-PEG-PLA) were prepared by using a simple emulsion-solvent evaporation technique. The nanoparticles were characterized for size, particle size distribution, surface charge, and encapsulation efficiency. Their cellular uptake and antiproliferative activity was evaluated against MCF-7 and MDA-MB-231 human breast cancer cells using flow cytometry and MTT assays, respectively. β-Sit-PLGA and β-Sit-PEG-PLA nanoparticles were spherical in shape with average particle sizes of 215.0 ± 29.7 and 240.6 ± 23.3 nm, a zeta potential of −13.8 ± 1.61 and −23.5 ± 0.27 mV, respectively, and with narrow size distribution. The encapsulation efficiency of β-Sit was 62.89 ± 4.66 and 51.83 ± 19.72 % in PLGA and PEG-PLA nanoparticles, respectively. In vitro release in phosphate-buffered saline (PBS) and PBS/with 0.2% Tween 20 showed an initial burst release, followed by a sustained release for 408 h. β-Sit-PLGA nanoparticles were generally stable in a protein-rich medium, whereas β-Sit-PEG-PLA nanoparticles showed a tendency to aggregate. Flow cytometry analysis (FACS) indicated that β-Sit-PLGA nanoparticles were efficiently taken up by the cells in contrast to β-Sit-PEG-PLA nanoparticles. β-Sit-PLGA nanoparticles were therefore selected to evaluate antiproliferative activity. Cell viability was inhibited by up to 80% in a concentration range of 6.64–53.08 μg/mL compared to the untreated cells. Taken together, encapsulation of β-Sitosterol in PLGA nanoparticles is a promising strategy to enhance its anticancer activity against breast cancer cells.

## 1. Introduction

Beta-sitosterol (β-Sit) is the most abundant plant phytosterol distributed in a wide variety of plant species [1,2]. Over the years, it has been reported in several studies to offer various health benefits—and most importantly, its inhibitory activity against a number of cancers. For example, β-Sit is reported to inhibit proliferation of breast cancer (MCF-7 and MDB-MB-231) [3,4], colon cancer [5,6], leukemia [7], and prostate cancer cells [8]. Interestingly, β-Sit modulates several disease targets by activating multiple signaling pathways, which would undoubtedly make it useful in treating such multifactorial and complex diseases as cancer. In summary, its anticancer activities are associated with the stimulation of apoptotic cell death [4], cell cycle arrest, and activation of the sphingomyelin cycle [8]. Despite its anticancer activities, β-Sit has largely remained a neglected natural product, mostly because of its lower in vitro efficacy as compared to other chemotherapeutic drugs [9]. 

As it is the case with several natural compounds, low aqueous solubility, which translates into low bioavailability, and coupled with low targeting efficacy, has limited clinical development of β-Sit. The use of nanoparticle drug delivery is the objective of several current studies aiming to enhance anticancer therapeutic efficacy of some natural compounds. In a recent review, several novel nanoformulations have been used to enhance the therapeutic potential of a large number of naturally derived drugs, such as curcumin, paclitaxel, doxorubicin, vincristine, camptothecin, artemisinin, resveratrol, honokoil, green tea catechins, and silymarin [10]. In fact, some of these natural drugs are already on the market, and others are undergoing clinical studies [10]. 

Little research has been done to enhance the therapeutic efficacy of β-Sit using drug delivery systems. Most studies conducted with β-Sit have focused on using it and its derivatives as an excipient to stabilize drug release or to enhance drug absorption [11,12,13,14,15]. However, in an earlier study, liposomal formulation containing β-Sit increased the natural killer cell activity and decreased colonies of metastatic B16BL6 melanoma cells in lungs of treated mice [16]. In other studies, cyclodextrins (CD) have been used to enhance the aqueous solubility and bioavailability of β-Sit. For example, several biological evaluations of β-Sit conducted by Awad et al. against a panel of cancer cells involved using 2-hydroxypropyl-β-cyclodextrin (HP-βCD) as a carrier vehicle [1,3,4,17]. These studies demonstrated that β-Sit inhibited cell proliferation at 16-32 μM concentration levels after 3–5 days of treatment. Generally, cyclodextrins have been used as excipients in pharmaceutical preparations to enhance solubility and bioavailability; however, the drug-CD complexes have been shown to have poor interaction with cell membranes, hence reducing bioavailability [18]. We hypothesized that encapsulating β-Sit in amphiphilic polymeric nanoparticles can offer better enhancement of anticancer activity against selected cancer-cell lines. This assumption is attributed to several beneficial properties of polymeric nanoparticles, such as enhanced drug-loading capacity, facilitated, controlled, and targeted drug release, and improved solubility of poorly soluble drugs [19].

PLGA and PEG-PLA are biocompatible, and in the case of PEG-PLA amphiphilic polymers, they have been investigated in several studies as drug delivery vehicles for hydrophobic drugs [20,21,22]. It is envisioned that PLGA or PEG-PLA can encapsulate β-Sit in the inner hydrophobic core, with the hydrophilic shell projecting in the aqueous environment [23]. This would consequently enhance the solubility of β-Sit in the aqueous phase. Besides this, the small size of the nanoparticles could potentially help to concentrate the drug at the disease site through the hypothesized enhanced permeability and retention (EPR) effect, hence enhancing its anticancer efficacy for several folds. 

## 2. Materials and Methods 

### 2.1. Materials

Resomer^®^ RG 502 H, Poly(d,l-lactide-*co*-glycolide) (PLGA) 50:50 (molecular weight 7 kDa) and mPEG-PLA diblock co-polymer (mPEG-PLA (R25) and mPEG-PLA (R45) were purchased from Evonik Nutrition & Care GmbH (Essen, Germany), Polyvinyl alcohol (PVA) Mw ~205 kDa), stigmasterol (95%), coumarin-6, and 2-Hydroxypropyl-beta-cyclodextrin (HP-β-CD) were purchased from Sigma-Aldrich (St. Louis, MO, USA). β-Sitosterol >70% was purchased from Cayman Chemicals (Ann Arbor, MI, USA), and Sulphuric acid (ACS grade) and acetic anhydride (AR) were purchased from Thermofisher Scientific (Waltham, MA, USA). All other solvents used were ACS-grade solvents.

Human breast adenocarcinoma cells (MDA-MB-231) and breast cancer cell line MCF-7 Dulbecco’s modified eagle’s medium (DMEM), Minimum essential medium (EMEM), and fetal bovine serum (FBS) were obtained from Sigma-Aldrich (St. Louis, MO, USA), and Trypsin 0.25% EDTA was purchased from Thermofisher Scientific (Waltham, MA, USA). All other chemicals were standard chemicals required for cell culture.

### 2.2. Analytical Method

The amount of β-Sit in solution was determined by a simplified calorimetric method based on the reaction of sterols with a solution of sulfuric acid in acetic anhydride (Liebermann-Burchard reagent) as described in previous studies [24,25,26,27,28,29,30]. Briefly, 80 mg of β-Sit were weighted and solubilized under stirring in 20 mL of dichloromethane (DCM). Working standard solutions were prepared by further dilution of the β-Sit stock in DCM. The absorbance of β-Sit in the standard solutions was measured at 622 nm, 10 min after adding Liebermann-Burchard reagent using a microplate reader with a quartz 96-well plate (FLUOstar Omega, BMG labtech, Ortenberg, Germany). A calibration curve was obtained by plotting absorbance versus the concentration of β-Sit standard solutions in DCM. The quantity of β-Sit, encapsulated or released from the nanoparticles, was determined according to the calibration curve prepared from standard solutions of β-Sit, but in this case after extraction of β-Sit with a known volume of dichloromethane (DCM). All experiments were carried out in triplicate. In each case, the presence of interfering substances in the samples that could affect the UV–vis spectrum of β-Sit was accounted for with the appropriate blank samples. All blank media had negligible absorption at 622 nm. 

### 2.3. Preparation of Nanoparticles

β-Sitosterol-loaded PLGA (β-Sit-PLGA) and β-sitosterol-loaded-PEG-PLA (β-Sit-PEG-PLA), as well as blank PLGA and PEG-PLA nanoparticles, were prepared by a simple emulsion-diffusion-solvent evaporation method [31]. The organic phase was prepared by dissolving PLGA and β-Sit in a volatile organic solvent. Then, the organic solution containing polymer and β-Sit was emulsified with a 2% solution of PVA in a ratio of 5:1 *v*/*v* (aqueous phase to organic phase) while vortex-mixing on high. The oil-in-water emulsion formed was further sonicated using a probe sonicator for 2 min to facilitate nanoparticle formation. The emulsion was stirred for 15 h using a magnetic stirrer to evaporate the organic solvent and to allow nanoparticles to harden. After complete evaporation of the organic solvent, nanoparticles were collected and washed three times with ultra-purified water by centrifugation (Eppendorf, centrifuge 5418R, Hamburg, Germany) at 16,900× *g* and 4 °C for 30 min. Then, the nanoparticles were suspended in 10 mL of previously filtered ultrapure water. Fluorescently labeled nanoparticles were prepared similarly, but in this case, 10 μL of coumarin-6 from a stock solution of 50 μg mL^−1^ was added to the organic phase containing 2 mg/mL of β-Sit and emulsified with 2% PVA. 

#### 2.3.1. Physico-Chemical Characterization of Nanoparticles

Particle size (Z-average), polydispersity index (PDI), and zeta potential of nanoparticles were measured using a Zetasizer (Nano ZS, Malvern Instruments, Malvern, UK). For particle size and PDI measurements, 100 μL of nanoparticle suspension was diluted tenfold in previously filtered ultrapure water. Another aliquot (100 μL) was diluted tenfold for zeta potential measurement. Results are expressed as mean ± standard deviation (*n* = 3). 

#### 2.3.2. Nanoparticle Surface Morphology

Surface morphology of the nanoparticles was measured using scanning electron microscopy (SEM) FEI, Helios G3 UC, (Thermofisher Scientific, Waltham, MA, USA). For sample preparation, 5 μL of the nanoparticle suspension was dropped onto filter paper attached to an adhesive carbon tape and mounted on aluminum stab. The SEM images were analyzed using the free software, ImageJ (NIH, Bethesda, MD, USA) to calculate particle size. 

#### 2.3.3. Encapsulation Efficiency and Percent Drug Loading

The amount of β-Sit encapsulated in nanoparticles was assayed by adapting a calorimetric method based on the reaction of sterols with a solution of sulphuric acid in acetic anhydride (Liebermann-Burchard reagent), as described in Section 2.2. Here, a fixed volume of the nanoparticle suspension was centrifuged for 30 min at 16,900× *g*. The nanoparticle pellet was then dissolved in DCM and absorbance was measured at 622 nm, 10 min after the addition of the Liebermann-Burchard reagent. This reaction time had been previously measured (Supplementary Figure S1b). Results are expressed as mean ± SD (*n* = 3). The amount of β-Sit encapsulated in the nanoparticle suspension was calculated from a standard curve prepared by a measuring absorbance of standard solutions of β-Sit (Supplementary Figure S2). Equations (1) and (2) were used to calculate encapsulation efficiency (EE) and drug-loading capacity [32].

(1) $$\mathrm{Encapsulation}\;\mathrm{efficiency}=\frac{Weight\;of\;\beta -Sit\;\left(mg\right)\;in\;nanoparticle\;suspension}{Weight\;of\;\beta -Sit\;used\;for\;formulation}\times 100$$

(2) $$\text{Drug-loading capacity}=\frac{Actual\;weight\;of\;\beta -Sit\;\left(mg\right)\;in\;nanoparticle\;suspension}{Theoretical\;weight\;of\beta -Sit\;+Theoretical\;weight\;of\;polymer}\times 100$$

### 2.4. In Vitro Release Profile 

In vitro release of β-Sit from β-Sit-PLGA and β-Sit-PEG-PLA nanoparticles was determined in PBS (PBS, 0.01 M, pH 7.4) and PBS with 0.2% Tween 20. Given the very low aqueous solubility of β-sitosterol, Tween 20 was included in the release medium to maintain sink conditions [33]. For each sample, 1.5 mL of the nanoparticle suspension corresponding to approximately 0.09 mg/mL of β-Sit was measured and centrifuged. The pellet was then re-suspended in 1.5 mL of PBS or PBS with 0.2% Tween 20. These samples were incubated at 37 ± 0.5 °C in a shaker (Mixer HC, Starlab international GmbH, Hamburg, Germany) mixing at 300 rpm. At preselected time intervals, the medium was centrifuged at 16,900× *g* for 10 min, and 1 mL of the supernatant was withdrawn for analysis. Subsequently, 1 mL of fresh release medium was added to replace the amount of release medium withdrawn. The withdrawn supernatant was extracted in a known volume of DCM for 30 min with occasional vortex mixing. The absorbance of the DCM extract was measured at 622 nm using a microplate reader (FLUOstar Omega, BMG Labtech). The amount of β-sitosterol released was determined according to the standard calibration curve prepared earlier.

### 2.5. Stability Study

Stability of the nanoparticles was studied as follows: nanoparticle suspensions in ultrapure water were stored at room temperature and under refrigerated conditions (4 °C). Particle stability was assessed by measuring size and PDI over a period of 30 days. On the other hand, stability of the nanoparticles in a biologically relevant milieu was determined by taking fixed volumes (1 mL) of the nanoparticle suspension, centrifuging at 16,900× *g* to pellet the nanoparticles, and re-suspending the pellet in phosphate-buffered saline (PBS, 0.01 M, pH 7.4) and PBS/FBS (50:50 *v*/*v*) and incubated at 37 °C for 24 h in a shaker [34,35]. At predetermined time intervals, the samples were centrifuged, and pellets were washed two times with ultrapure water. The purified pellets were then re-suspended in ultrapure water and characterized as described above for size, PDI, and surface morphology.

### 2.6. Biological Evaluation of Nanoparticles

Two breast cancer cells, MCF-7 and MDA-MB-231, representing hormone-dependent and hormone-independent stages of human breast carcinoma, were used to study cytotoxicity and the cellular uptake of nanoparticle formulations, as described below.

#### 2.6.1. Cell Culture

MCF-7 cells were cultured in EMEM, supplemented with 10% FBS, 1% penicillin/streptomycin, 2 mM L-glutamine, and 1% non-essential amino acids in a humified environment at 37 °C and 5% CO_2_. On the other hand, MDA-MB-231 cells were grown in Dulbecco-modified eagle’s medium (DMEM) supplemented with 10% FBS and 1 % penicillin/streptomycin in a humidified incubator at 37 °C and 5% CO_2_.

#### 2.6.2. Cellular Uptake by Flow Cytometry

Considering that β-Sit is a non-fluorescent molecule, coumarin-6 was incorporated as a fluorescent dye during nanoparticle formulation to study the cellular uptake of β-Sit-PLGA and β-Sit-PEG-PLA. Aliquots of MDA-MB-231 or MCF-7 cells (5 × 10^4^ cells) were seeded in 24-well plates in triplicate and incubated at 37 °C for 24 h. The cells were then transfected with 100 μL of courmarin-6-labeled β-Sit-PLGA (β-Sit-PLGA-C6) and coumarin-6 labeled β-Sit-PEG-PLA (β-Sit-PEG-PLA-C6) nanoparticles. After predetermined time periods, the cell medium was discarded, and cells were trypsinized and washed with cold PBS twice. The cells were then examined with a flow cytometer (Attune NxT flow cytometer, Thermofisher Scientific, Waltham, MA, USA) at an excitation wavelength of 488 nm and detection fluorescence of 525 nm. Data were collected for 10,000 gated events per sample [36]. 

#### 2.6.3. Confocal Microscopy

MCF-7 and MDA-MB-231 cells were seeded at a density of 2 × 10^4^ cells per well in 8-well chamber slides and incubated at 37 °C for 24 h. The cells were washed with sterile PBS and treated with coumarin-6 labeled β-Sit-PLGA nanoparticles and incubated for 4 h. To examine the cellular internalization of nanoparticles, cells were further incubated with Lyso Tracker^TM^ red, which is a fluorescent organelle-specific contrasting dye. After one hour of incubation, the cells were washed thrice with sterile PBS and fixed with 4% paraformaldehyde (PFA) solution (200 μL) per chamber and incubated for 15 min. Cells were then washed with sterile PBS and incubated for another 20 min after staining with DAPI (4′,6-diamidino-2-phenylindole) at a final concentration of 1 μg/mL (200 μL per chamber). The cells were finally washed with PBS twice and mounted using Fluorsave^TM^ for confocal microscopy. Fluorescence images were acquired using a laser scanning confocal microscope (Leica SP8 inverted, Software: LAS X, Leica microsystems GmbH, Wetzlar, Germany). The following were the image acquisition parameters: DAPI channel excitation at 405 nm, emission at 420–520 nm while Coumarine 6 was excited 488 nm, emission wavelength was set at 490-550 nm, and the Lysotracker red excitation wavelength was 552 nm, with emission at 600–730 nm.

#### 2.6.4. In Vitro Antiproliferative Activity

The effect of β-Sit-PLGA nanoparticles on cell proliferation was tested using the (3-(4,5-Dimethylthiazol-2-yl)-2,5-Diphenyltetrazolium Bromide) MTT calorimetric assay in MDA-MB-231 and MCF-7 cells [27]. Cells were seeded at a density of 1 × 10^4^ cells per well in a 96-well plate, and incubated for 24 h. The cells were treated at varying concentrations (53.08, 26.24, 13.12, and 6.64 μg/mL of β-Sit in nanoparticle suspension, respectively) of β-Sit-PLGA nanoparticles and incubated for 24 h. The medium was removed and MTT solution (10 μL) was added and incubated for 4 h. The purple formazan crystals were dissolved in dimethyl sulfoxide (DMSO) (200 μL) and incubated for 10 min to allow complete dissolution of the dye. Absorbance was measured at 570 nm using a microplate reader. Control groups included cells treated with blank nanoparticles at equivalent concentrations as the treatment groups (6.64–53.08 μg/mL) and untreated cells. 

### 2.7. Statistical Analysis

All results are expressed as mean ± standard deviation. Statistical differences between groups were evaluated by one-way or two-way ANOVA and Bonferroni post-tests using a Graphpad Prism, version 5.00, GraphPad Software, La Jolla, CA, USA, 2007. *P* < 0.05 was considered statistically significant, where applicable.

## 3. Results and Discussions

### 3.1. Analytical Methods

A calorimetric method based on the reaction of sterols with acetic anhydride and concentrated sulphuric acid was evaluated to assay β-Sit in a nanoparticle matrix. The method was evaluated for precision, linearity, accuracy, limit of detection, and limit of quantitation. The linearity of the response was verified over a concentration range of 2–0.0125 mg/mL (*R*^2^ = 0.9966) (Supplementary Figure S1). The limit of detection (LOD) (estimated as three times the background noise) was 7.43 μg/mL. The limit of quantitation (LOQ) (estimated as 10 times the background noise) was 22.0 μg/mL. The % root mean square deviation (RSD) for inter-assay and intra-assay precision were in the range of 0.919 to 2.986, suggesting that the analytical method gives reproducible results (Supplementary Table S1). The method’s recovery falls within the range of 95.45 to 103.60%, suggesting that the accuracy of the method is good and that there is little interference of the nanoparticle matrix on the extraction of β-Sit (Supplementary Table S2). 

### 3.2. Formulation Studies

Preliminary optimization studies were conducted in order to prepare β-Sit-PLGA and β-Sit-PEG-PLA nanoparticles with the desired physicochemical characteristics. Here, the effect of different nanoparticle formulation process parameters on particle size (Z-average), size distribution, surface charge, and drug-loading efficiency were studied. Process and formulation parameters investigated included the organic solvent, sonication amplitude, duration of solvent evaporation, concentration of emulsifier, aqueous-to-organic phase ratio, amount of polymer, and the feeding amount of drugs used. The approach adapted involved varying the formulation parameters one at a time along with varying sonication power amplitude, while keeping other parameters constant. First, the effect of these process and formulation parameters were studied using blank nanoparticles (Supplementary Table S3). Optimal parameters were selected to prepare stigmasterol-loaded PLGA nanoparticles (Supplementary Table S4) and later, the optimized parameters were adapted to fabricate β-Sit-PLGA and β-Sit-PEG-PLA nanoparticles. Stigmasterol was chosen as a model for optimization studies due to its similar structure with β-sitosterol; however, it is cheaper and readily available. 

**Figure 1:**
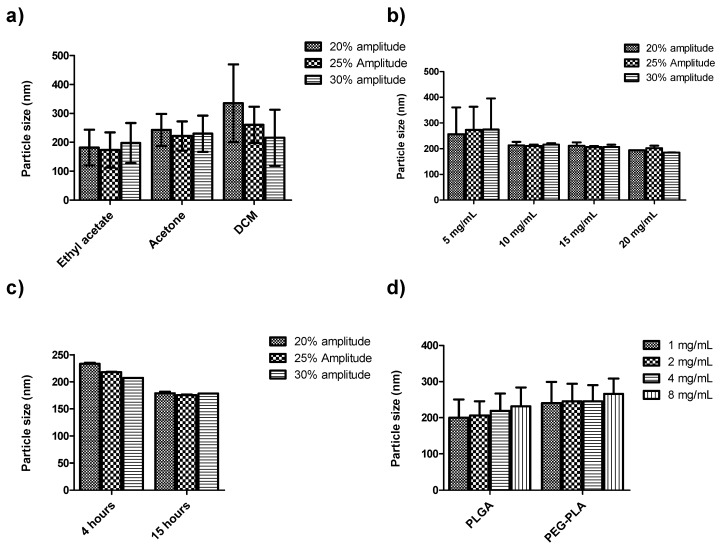
Particle size variation of blank nanoparticles (**a**–**c**) and stigmasterol-loaded nanoparticles (**d**) under different process and formulation parameters: (**a**) effect of organic solvent; (**b**) effect of polymer concentration; (**c**) effect of duration of solvent evaporation; and (**d**) effect of feeding-drug concentration. Results represent mean ± SD (*n* = 3).

Particle sizes were smallest when ethyl acetate was used as the organic solvent compared to acetone and dichloromethane (DCM), respectively (Figure 1a). This effect can be explained by the partial miscibility of ethyl acetate in water, and therefore by the low interfacial tension which exists between water and ethyl acetate, leading to the formation of smaller nanoparticles [37]. On the other hand, DCM is immiscible with water, and this creates a very high interfacial tension between the aqueous and organic phases, leading to the formation of bigger-size nanoparticles. Song et al. observed that high water immiscibility of DCM resulted in the formation of aggregates with particle sizes up to 390 nm [38]. We similarly observed aggregates when DCM was used as the organic solvent. Varying sonication amplitudes from 20–30% did not have a significant effect on particle size under different formulation parameters (Figure 1). The duration of solvent evaporation is one important process parameter that can affect nanoparticle size. Shorter solvent evaporation duration leads to the formation of bigger-size particles compared to those of longer solvent evaporation time [39]. Our preliminary investigations indicate that nanoparticles were rather smaller when the organic solvent was evaporated for a longer time (15 h) compared to 4 h of solvent evaporation time (Figure 1c). On the other hand, there was no significant variation in particle size of blank nanoparticles by changing the polymer concentration from 5–20 mg/mL along with the sonication power amplitude (*P* > 0.05) (Figure 1b). From these results, 20 mg/mL of PLGA or PEG-PLA and 2% *w*/*v* of PVA were chosen to further optimize the formulation of stigmasterol-loaded PLGA and PEG-PLA nanoparticles. The nanoparticles were homogenized at 30% sonication power amplitude, and the organic solvent was evaporated for 15 h with magnetic stirring. Here, as the feeding drug weight increased from 1 to 8 mg/mL, the size of the nanoparticles gradually increased (Figure 1d). Similar observations were made in previous studies; for example, nanoparticle size was shown to increase drastically on increasing feeding drug content of curcumin and budesonide [12,23]. At a constant polymer-to-drug feeding ratio, a fixed amount of the drug was expected to be incorporated. A further increase in drug feeding amount increased the viscosity of the nanoparticle matrix, thus leading to bigger-size particles [39]. Drug feeding concentrations of 2 and 4 mg/mL were therefore selected to further study drug encapsulation efficiency, using two approaches of preparing the organic phase. In one approach (A), an organic solution of the polymer was added to the drug powder and stirred for 30 min to dissolve the drug. The other variation (B) involved making an organic solution of polymer and β-Sit separately and then mixing the two organic solutions by magnetic stirring for 30 min. As shown in the supplementary information, drug encapsulation efficiency was higher using approach (B) than using approach (A) (Supplementary Table S4). Generally, results of the preliminary studies show that the average particle size was 218.5 ± 36.7 nm (range of 173.5–335.7 nm) with a narrow size distribution (PDI = 0.115 ± 0.06, range 0.004–0.28) and negative surface charge (Zeta potential −12.15 ± 3.82 mV, range −7.97 to −24.6 mV). Based on these results, 20 mg/mL of polymer, 2% PVA, 2–4 mg/mL of drug feeding concentration, and 30% sonication amplitude were selected for the fabrication of β-Sit-PLGA, β-Sit-PEG-PLA nanoparticles with 15 h of solvent evaporation while stirring.

### 3.3. Optimization Study for the Encapsulation of β-Sit

**Figure 2:**
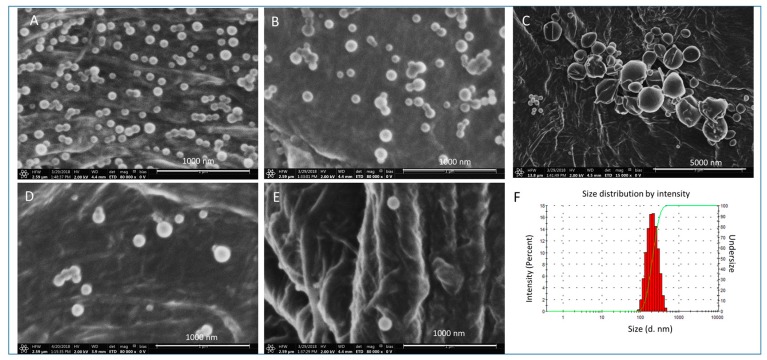
Scanning electron microscope (SEM) micrographs of nanoparticles: (**A**) Blank poly(lactide-*co*-glycolic acid) (PLGA) nanoparticles; (**B**) β-Sit-PLGA-EtAC; (**C**) β-Sit-PLGA-DCM; (**D**) Blank poly(ethylene glycol)-block-poly(lactic acid) (PEG-PLA); and (**E**) β-Sit-PEG-PLA nanoparticles. (**F**) Representative particle size and size distribution, as measured using dynamic light scattering (DLS).

β-Sit-PLGA and β-Sit-PEG-PLA nanoparticles were prepared by choosing the following optimized process and formulation parameters: 2–4 mg/mL drug feeding concentration, 5:1 *v*/*v* aqueous-to-organic phase ratio, 30% sonication power amplitude, and 15 h of solvent evaporation time. Ethyl acetate and DCM were used for PLGA-based formulations (herein labeled as β-Sit-PLGA-EtAC and β-Sit-PLGA-DCM, respectively). While acetone was adapted for PEG-PLA-based formulations (herein labeled as β-Sit-PEG-PLA (R25) and β-Sit-PEG-PLA (R45) to signify the type of polymer, respectively), the particles were characterized according to their size, polydispersity index, zeta potential, and morphology. Table 1 shows the variation of particle size, PDI, and zeta potential of the nanoparticles. Nanoparticle size increased as the drug feeding concentration was doubled. Similar to the previous observations in preliminary experiments, β-Sit-PLGA-EtAC nanoparticles were smaller, with narrow particle-size distribution (PDI < 0.2) compared with β-Sit-PLGA-DCM and β-Sit-PEG-PLA nanoparticles (Table 1). Moreover, β-Sit-PLGA-EtAC and β-Sit-PLGA-DCM nanoparticles had slightly lower negative surface charge, compared with β-Sit-PEG-PLA nanoparticle formulations. It is suspected that the amount of PEG in PEG-PLA used in this study was insufficient to neutralize the surface charge of the PEG-PLA nanoparticles. X-ray photoelectron spectroscopy measurements showed that the surface coverage of PEG chain in PEG-PLA nanoparticles was only 21.11% (ζ = −13.8 mV), compared to 63.73% in PEG-PLA-PEG nanoparticles (ζ = −2.23 mV) [40]. In other similar studies, the surface charge of PEG-PLGA nanoparticles was comparable to non-PEGylated PLGA nanoparticles (−26.7 compared to −20.9 mV) [20]. Since PEGylation increases hydration of nanoparticles, this could have resulted in higher surface charge due to consequent hydrolysis [20]. Unfortunately, β-Sit-PLGA-DCM showed a tendency to aggregate. The scanning electron micrographs show that the particles were spherical in shape with smooth surfaces (Figure 2A–E), and confirmed the aggregation tendency of β-Sit-PLGA-DCM (Figure 2C). On the other hand, physicochemical properties of coumarin-6-labeled nanoparticles did not vary significantly from those of non-fluorescent nanoparticles, shown in Table 1b.

### 3.4. Encapsulation Efficiency and Drug-Loading Capacity

Table 2 shows the variation in encapsulation efficiency and drug-loading capacity of β-Sit-PLGA and β-Sit-PEG-PLA formulations at 2 and 4 mg/mL drugfeeding concentrations. In the statistical sense, there is no significant difference in encapsulation efficiency and drug-loading capacity at 2 mg/mL and 4 mg/mL drug feeding concentrations for all the formulations tested (*P* > 0.05). Therefore, formulations with 2 mg/mL of β-Sit feeding concentrations were selected for further investigation. 

### 3.5. In Vitro Drug Release Profile

**Figure 3:**
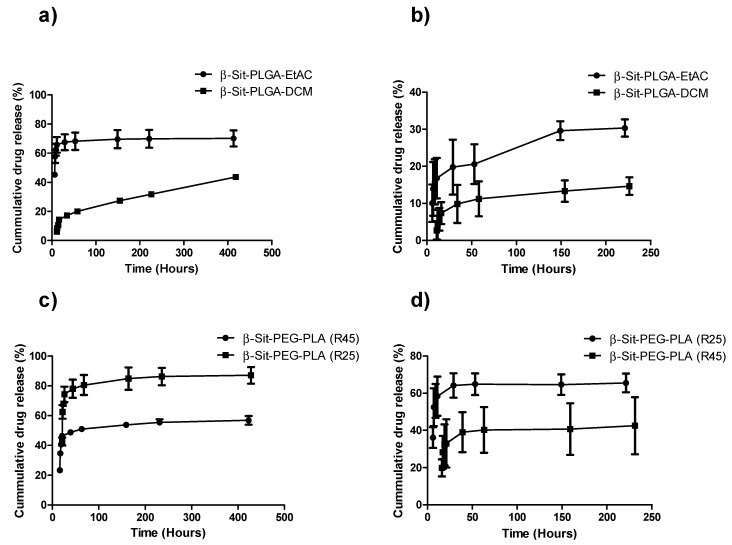
Cumulative release of β-Sit from β-Sit-PLGA and β-Sit-PEG-PLA nanoparticle formulations in (**a**) β-Sit-PLGA PBS/0.2% Tween 20, (**b**) β-Sit-PLGA in PBS, (**c**) β-Sit-PEG-PLA in PBS/0.2% Tween 20, and (**d**) β-Sit-PEG-PLA in PBS, respectively. Nanoparticles were suspended in PBS with 0.2% Tween 20 and PBS alone, and incubated at 37 °C in a shaker. The amount of β-Sit released was determined by a UV-Vis calorimetric assay, as described. Each sample was analyzed in triplicate. Results represent mean ± SD (*n* = 3).

The in vitro release of nanoparticles was studied in PBS and PBS/Tween 20 media. As shown in Figure 3, there was an initial burst release for the first 24 h, followed by a very slow and sustained release of β-Sit for up to 408 h (17 days) in PBS/Tween 20. The cumulative release accounts for 70.10 ± 5.50, 87.11 ± 5.54, and 56.83 ± 2.99% of β-Sit were released from β-Sit-PLGA-EtAC, β-Sit-PEG-PLA (R25), and β-Sit-PEG-PLA (R45) in PBS/Tween 20, respectively (Figure 3a,c). Interestingly, β-Sit-PLGA-DCM nanoparticles exhibited slow gradual drug release in PBS/Tween 20, with only 43.61 ± 0.80% of β-Sit released after 408 h (Figure 3a). Similar drug release profiles were found for both formulations, that is, β-Sit-PEG-PLA (R25) and β-Sit-PEG-PLA (R45) in PBS. Generally, the initial burst release is attributed to the drug molecules adsorbed on the nanoparticle surface and those poorly entrapped in the nanoparticle matrix. Slow sustained release is attributed to the diffusion of drug molecules entrapped in the nanoparticle core. However, there is a more restricted release of β-Sit from β-Sit-PLGA-EtAC and β-Sit-PLGA-DCM formulations in PBS, with only 30.33 ± 3.28 and 14.66 ± 3.36% released in PBS after 216 h (Figure 3b). To a certain extent, this release pattern is attributed to the bigger size of β-Sit-PLGA-DCM particles, which increases the drug’s diffusional path length [41]. In addition to particle size, the low solubility of β-Sit in PBS is expected to be the main cause for the slow release in PBS. 

### 3.6. Stability

**Figure 4:**
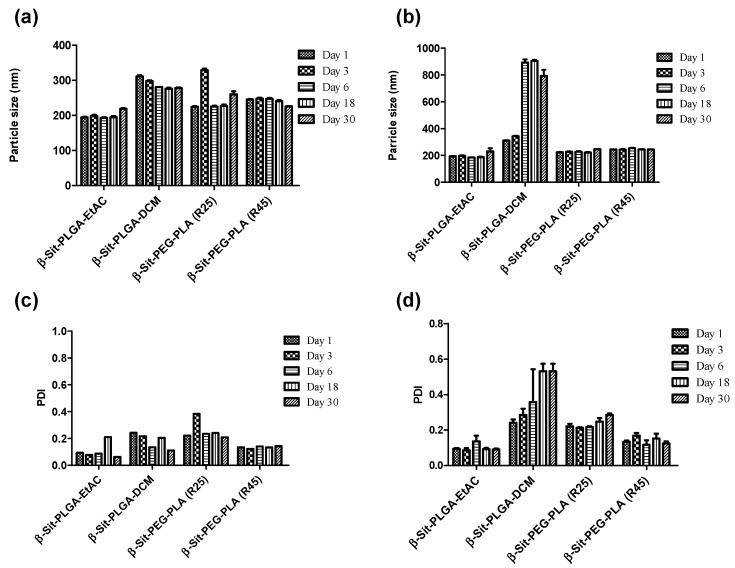
Storage stability of nanoparticles at room temperature and 4 °C. Nanoparticles were suspended in previously filtered ultrapure water and stored at room temperature and at 4 °C in the dark for 30 days. Stability was characterized by measuring particle size (**a**,**b**) and polydispersity index (PDI) (**c**,**d**) at room temperature and 4 °C, respectively. Results represent mean ± SD (*n* = 3).

Stability of the nanoparticles was investigated at room temperature and at 4 °C. Stability was characterized by measuring particle size and PDI at five time-points over a period of 30 days. Figure 4 shows the variation in particle size and PDI of the particles. At room temperature, the size of nanoparticles remained fairly uniform with monomodal particle distribution (PDI < 0.2, Figure 4a,c). Slight particle aggregation was only observed with β-Sit-PLGA-DCM nanoparticles. Similarly, nanoparticle suspensions were stable when stored at 4 °C, except for β-Sit-PLGA-DCM particles which strongly aggregated after the third day (Figure 4b,d). β-Sit-PEG-PLA (R25) particles also showed signs of aggregation at 4 °C (PDI >2, Figure 4d). Particles with aggregation tendency (β-Sit-PLGA-DCM and β-Sit-PEG-PLA (R25)) were therefore not considered for any subsequent investigations. 

**Figure 5:**
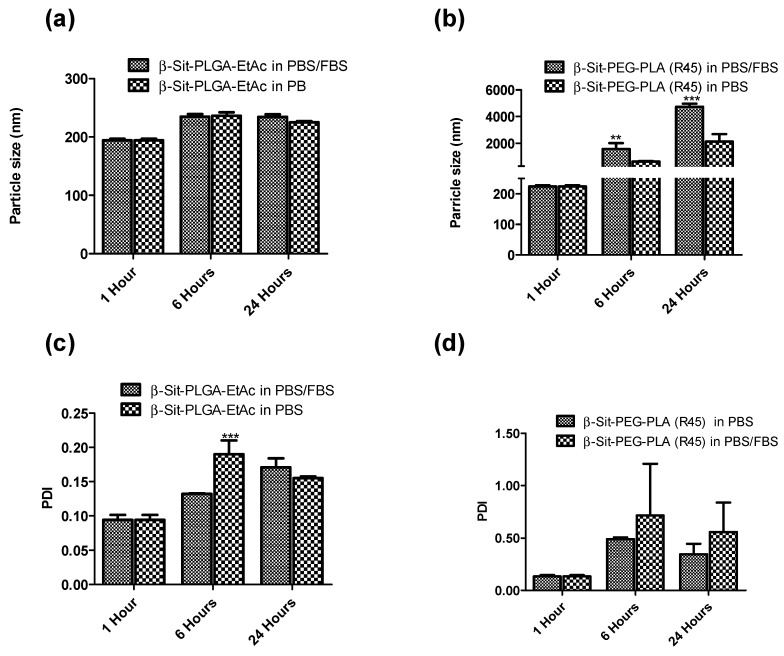
Stability of β-Sit-PLGA-EtaC and β-Sit-PEG-PLA (R45) nanoparticle suspension in PBS and PBS/fetal bovine serum (FBS) (50:50 *v*/*v*) at 37 °C. Nanoparticles were suspended in 1.5 mL of PBS or PBS/FBS and stored at 37 °C in a shaker for 24 h. Variation in particle size (**a**,**b**) and PDI (**c**,**d**) were measured at three time points over a 24-hour period. Results represent the mean ± SD of measurements taken in triplicate (*n* = 3). (*** *P* < 0.001, ** *P* < 0.01).

β-Sit-PLGA-EtAC and β-Sit-PEG-PLA (R45) were further selected for stability study in a protein-rich medium to assess their in vivo stability. In this case, the particles were suspended in PBS and PBS/FBS at 37 °C for 24 h in a shaker. Stability was characterized similarly by measuring particle size and particle distribution at three time-points. β-Sit-PLGA-EtAC nanoparticles maintained uniform particle sizes and narrow particle-size distribution (PDI < 0.2, Figure 5a). The surface morphology of the nanoparticles is presented in Figure S3 (Supplementary Materials). On the contrary, β-Sit-PEG-PLA (R45) nanoparticles aggregated during a 24 h incubation in PBS and PBS/FBS (Figure 5b). Given the very high negative surface charge of β-Sit-PEG-PLA nanoparticles, it is evident that the positively charged components in FBS caused the aggregation of these nanoparticles. Although PEGylation is expected to shield the surface of PEG-PLA nanoparticles [20], it seems that in this case the percentage of PEG was insufficient to offer protection to prevent aggregation. 

### 3.7. Cellular Uptake by Flow Cytometry

**Figure 6:**
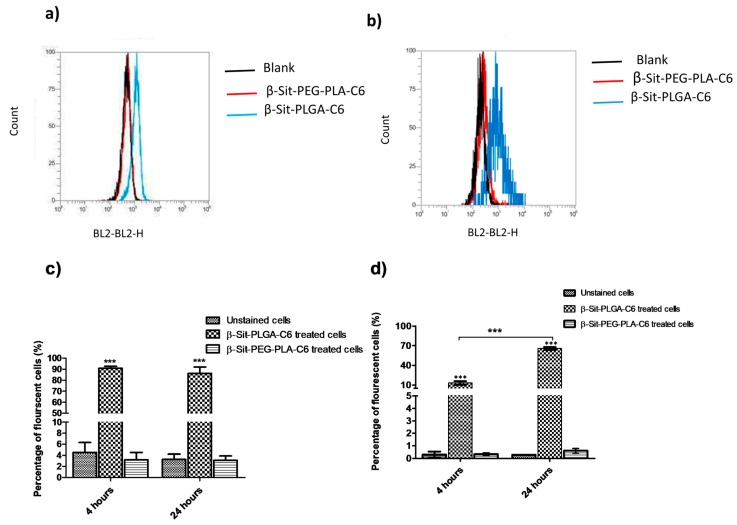
Flow cytometry distribution of the fluorescence intensity of coumarin-6 after (**a**) MDA-MB-231 cells and (**b**) MCF-7 cells were incubated for 24 h with β-Sit-PLGA-C6 and β-Sit-PEG-PLA-C6 nanoparticles, respectively, and the mean fluorescence intensity of coumarin-6 after (**a**) MDA-MB-231 cells and (**b**) MCF-7 cells were incubated for 24 h with β-Sit-PLGA-C6 and β-Sit-PEG-PLA-C6 nanoparticles, respectively. The mean fluorescence intensity of coumarin-6 after MDA-MB-231 (**c**) and MCF-7 (**d**) cells were incubated with β-Sit-PLGA-C6 and β-Sit-PEG-PLA-C6 for 4 and 24 h, respectively. Untreated cells were used as controls (*n* = 3), (*** *P* < 0.001).

The cellular internalization of nanoparticles was studied quantitatively, using flow cytometry in MDA-MB-231 and MCF-7 cells. Coumarin-6 is a fluorescent compound that has been used in other investigations to study nanoparticle uptake [40]. Courmarin-6 was incorporated into β-Sit-PLGA and β-Sit-PEG-PLA nanoparticles during formulation. Results of fluorescence-activated cell sorting (FACS) analysis, indicating the proportion of fluorescent cells relative to the control, are presented in Figure 6. The results exhibit that β-Sit-PLGA-C-6 nanoparticles were internalized by MDA-MB-231 cells over a 24 h incubation time with maximum uptake observed four h post incubation, relative to the untreated cells (Figure 6c,d). β-Sit-PEG-PLA-C-6 nanoparticles were poorly internalized by MDA-MB-231 cells with respect to the untreated cells (*P* < 0.001). On the other hand, β-Sit-PLGA-C6 nanoparticles were taken up by MCF-7 cells in a time-dependent manner, with the highest proportion of fluorescent cells observed 24 h post-incubation. Similar as in MDA-MB-231 cells, β-Sit-PEG-PLA-C6 nanoparticles were poorly taken up by MCF-7 cells. Several factors, such as nanoparticle size, shape, surface charge, and surface modification affect the cellular uptake of nanoparticles [42]. Beyond these, extrinsic factors, such as the culture medium in which the nanoparticles are suspended, affect the bio-fate of nanoparticles. β-Sit-PLGA, as well as coumarin-6 labeled nanoparticles, generally had smaller particle sizes (Z-average 215 nm) compared to β-Sit-PEG-PLA nanoparticles (Z-average 240 nm). This could have led to the efficient uptake of β-Sit-PLGA compared to β-Sit-PEG-PLA nanoparticles. Moreover, stability evaluation in PBS and PBS/FBS indicates that β-Sit-PLGA nanoparticles maintained uniform and small particle sizes and narrow size distributions as compared to β-Sit-PEG-PLA nanoparticles after 24 h of incubation (Figure 5). Additionally, β-Sit-PLGA particles have less negative surface charge compared to β-Sit-PEG-PLA nanoparticles. Shan et al. observed that the proportion of fluorescently labeled cells decreased significantly when treated with coumarin-6 labeled mPEG-PLA and PEG-PLA-mPEG nanoparticles compared to PLA nanoparticles [40]. It can be inferred at this point that the culture medium formed a bio-corona around β-Sit-PEG-PLA-C6 nanoparticles, which caused particle aggregation preventing their uptake by the cells, whereas this was not the case for β-Sit-PLGA-C6 nanoparticles.

### 3.8. Confocal Microscopy

**Figure 7:**
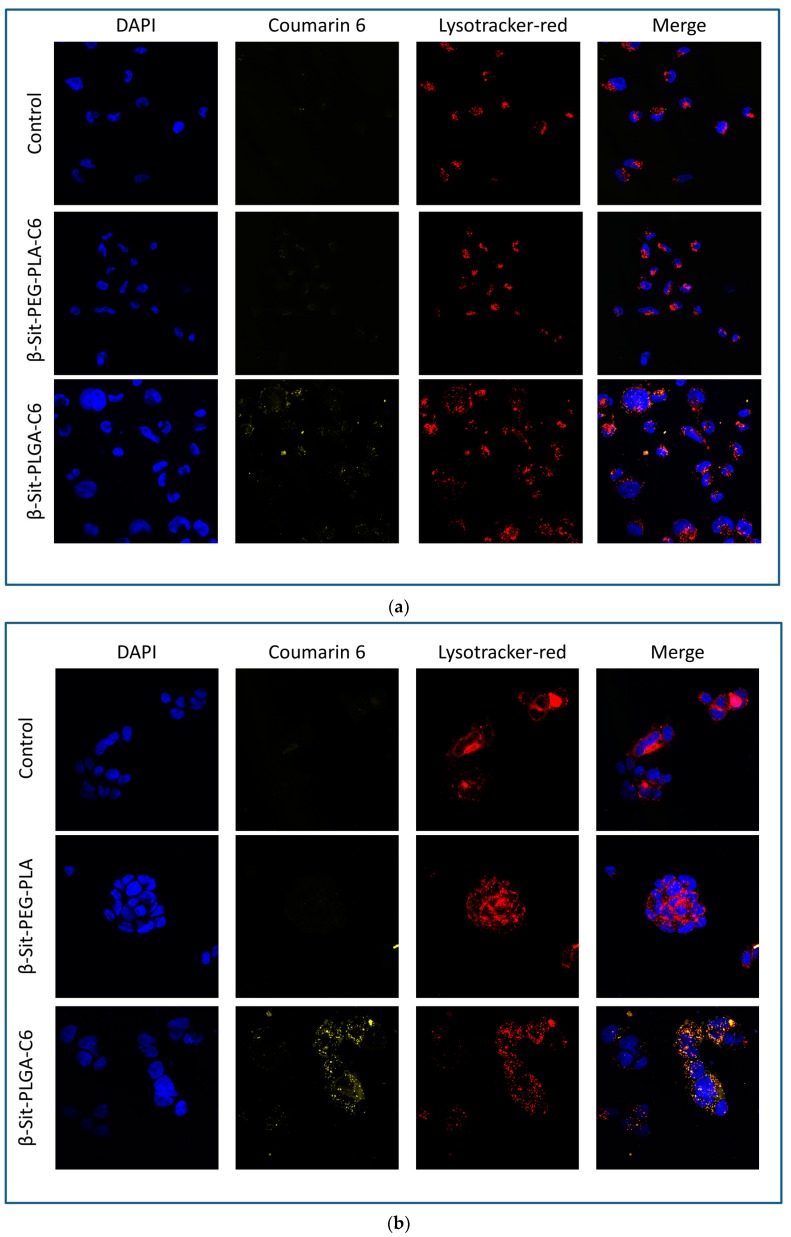
Fluorescence images of MDAMB-231 (**a**) and MCF-7 (**b**) cells after treatment with coumarin-6-labeled B-Sit-PLGA and B-Sit-PEG-PLA nanoparticles.

Figure 7 exhibits the fluorescence images by laser scanning confocal microscopy of coumarin-6-labeled β-Sit-PLGA and PEG-PLA nanoparticles in MDA-MB-231 and MCF-7 cells, after 4 h of incubation. Control cells treated with β-Sit-PEG-PLA did not show any green fluorescence, indicating that β-Sit-PEG-PLA nanoparticles were not internalized by the cells. Meanwhile, cells treated with β-Sit-PLGA-C6 showed green fluorescence, suggesting internalization of Sit-PLGA-C6 nanoparticles (Figure 7a,b) by MDA-MB-231 and MCF-7 cells, respectively. These results are in conformity with the flow cytometry results. Poor uptake of β-Sit-PEG-PLA-C6 is attributed to big particle size. Incorporating coumarin-6 into the nanoparticles resulted in a slight increase in particle size, which may as well have affected nanoparticle uptake by the cells.

### 3.9. In Vitro Antiproliferative Assay

**Figure 8:**
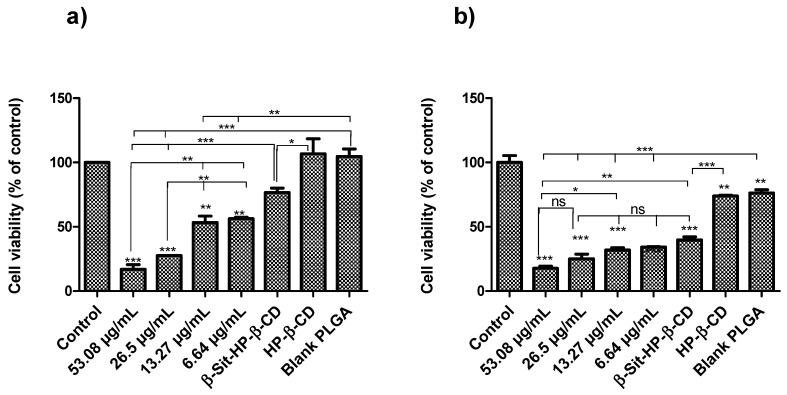
Cell viability of (**a**) MDA-MB-231 and (**b**) MCF-7 cells when incubated with β-Sit-PLGA nanoparticles. Cells were seeded at a density of 1 × 10^4^ cells per well and treated with varying concentrations of nanoparticle suspension and appropriate controls. Cell viability is expressed as a percentage of the control (untreated cells). Cell viability for blank PLGA treatments represents an average of all treatments at 6.64–53.08 μg/mL of PLGA. Results represent mean ± SD (*n* = 3) (*** *P* < 0.001, ** *P* < 0.01, * *P* < 0.05, ns, no statistical difference).

MTT assay is one of the common assays used to study cell viability and proliferation. It depends on the ability of viable cells to reduce the yellow MTT dye to insoluble purple formazan crystals [25,27]. Here, MTT assays were used to study the antiproliferative activity of β-Sit-PLGA nanoparticles. Since β-Sit-PEG-PLA nanoparticles showed very poor cellular uptake according to the FACS analysis, they were not included in the cell viability study. As shown in Figure 8, cell viability decreased overall for both cell lines in a dose-dependent manner, with about 80% reduction at the highest concentration levels of the treatment groups (26.5–53.08 μg/mL) over a 24 h incubation period (* *P* < 0.05). Inhibition of cell viability at lower concentration levels (6.64 and 13.27 μg/mL) was comparable to that of free β-sitosterol (13.27 μg/mL) in a HP-β-CD carrier. Blank PLGA nanoparticles at concentrations equivalent to the treatment groups (6.64–53.08 μg/mL) were also evaluated for any inherent cytotoxicity against the test cells. The results indicate that there was no significant difference in cell viability for cells treated with blank PLGA nanoparticles at the equivalent concentrations of β-Sit-PLGA; thus, the results are presented as an average for the blank treatments. Awad et al. observed that β-sitosterol inhibited the proliferation of MDA-MB-231 cells by 66% and 80% after 3 and 5 days of incubation, respectively [3]. Our results show that the same inhibition of cell proliferation is achieved in 24 h of incubation, suggesting that the nanoparticle formulation offers an advantage that enables β-Sit-PLGA particles to be easily concentrated at the disease site. It is believed that β-Sit is practically insoluble at higher concentration levels, thus limiting its activity [1]. In this regard, the nanoparticle formulation increases the solubility of β-Sit and makes it more available to the cells, thereby enhancing its antiproliferative activity, especially at higher concentration levels. 

## 4. Conclusions

In this study, PLGA and PEG-PLA were successfully utilized to encapsulate β-sitosterol. The particle size of the optimized β-Sit-PLGA formulations was approximately 215 nm on average, with a narrow size distribution and slightly low negative surface charge (ca. −13 mV) compared to β-Sit-PEG-PLA-based nanoparticles. An in vitro release study indicates biphasic release kinetics with an initial burst release, whereafter the drug release was sustained. From the FACS analysis, small size and low surface charge facilitated the easy uptake of β-Sit-PLGA nanoparticles compared to β-Sit-PEG-PLA nanoparticles. This suggests PLGA to be a better polymer for encapsulation of β-Sit compared to PEG-PLA. Encapsulation of β-Sit into PLGA nanoparticles enhanced its antiproliferative activity against MCF-7 and MDA-MB-231 cells in a concentration-dependent manner relative to the control groups. The results of this study demonstrate that encapsulation of β-Sit into PLGA nanoparticles could be a promising strategy to enhance its therapeutic efficacy against cancer. 

## Figures and Tables

**Table 1:** Particle size, polydispersity index, and zeta potential of (**a**) β-Sit-PLGA and β-Sit-PEG-PLA nanoparticles, and (**b**) coumarin-6-labeled nanoparticles. Results represent mean ± SD (*n* = 3).

**(a)**						
**Formulation**	**Weight of β-sitosterol Used for Formulation**					
	**2 mg/mL**			**4 mg/mL**		
	**Z-average (nm)**	**PDI**	**ζ (mV)**	**Z-average (nm)**	**PDI**	**ζ (mV)**
β-Sit-PLGA-EtAC	215.1 ± 29.7	0.10 ± 0.003	−13.9 ± 1.61	231.2 ± 0.60	0.126 ± 0.02	−16.1 ± 7.09
β-Sit-PLGA-DCM	311.1 ± 94.4	0.242 ± 0.03	−14.0 ± 6.01	261.6 ± 4.74 ^a^	0.113 ± 0.03 ^a^	−16.5 ± 0.21 ^a^
β-Sit-PEG-PLA (R45)	240.6 ± 23.3	0.18 ± 0.05	−23.5 ± 0.27	276.7 ± 1.01	0.197 ± 0.01	−21.5 ± 6.56
β-Sit-PEG-PLA (R25)	239.5 ± 7.9	0.17 ± 0.05	−24.5 ± 0.99	279.4 ± 0.85	0.112 ± 0.04	−21.2 ± 7.01
**(b)**						
**Formulation**	**Physicochemical Properties of Coumarin 6-Labeled Nanoparticles**					
	**Z-average (nm)**		**PDI**		**ζ (mV)**	
β-Sit-PLGA-EtAC	228 ± 0.45		0.10 ± 0.01		−16.8 ± 0.33	
β-Sit-PEG-PLA (R45)	245 ± 0.66		0.09 ± 0.02		−19.6 ± 0.22	

^a^ Result represents mean ± SD (*n* = 2); ζ zeta potential.

**Table 2:** Encapsulation efficiency and drug-loading capacity of β-Sit-PLGA and β-Sit-PEG-PLA nanoparticles. Results represent mean ± SD (*n* = 3).

Formulation	Weight of β-sitosterol Used for Formulation					
	2 mg/mL			4 mg/mL		
	EE (%)	EDL %	ADL (%)	EE (%)	EDL %	ADL (%)
β-Sit-PLGA-EtAC	62.89 ± 4.66	4.76	3.00 ± 0.22	48.41 ± 23.82	9.09	4.4 ± 2.17
β-Sit-PLGA-DCM	85.13 ± 6.35	4.76	4.05 ± 0.30	88.48 ± 0.01 ^a^	9.09	8.04 ± 0.01 ^a^
β-Sit-PEG-PLA (R45)	51.83 ± 19.72	4.76	2.47 ± 0.94	71.02 ± 22.48	9.09	6.46 ± 2.04
β-Sit-PEG-PLA (R25)	34.84 ± 1.71	4.76	1.67 ± 0.08	66.85 ± 8.13	9.09	6.08 ± 0.74

EE = encapsulation efficiency, EDL = expected drug loading, ADL = actual drug loading. ^a^ Result represents mean ± SD (*n* = 2).

## Supplementary Materials

The following are available online at http://www.mdpi.com/1999-4923/10/4/232/s1, Figure S1: Time course for calorimetric reaction of β-sitosterol with Liebermann-Burchard reagent. Figure S2: UV-Vis spectrum for calorimetric reaction of β-sitosterol with Liebermann-Burchard reagent. Figure S3: Surface morphology of nanoparticles after incubation in PBS and PBS/FBS. Table S1: Method precision characterized by intra-assay and inter-assay precision. Table S2: Spike recovery for determination of Liebermann-Burchard method for determination of β-Sit. Table S3: Physicochemical properties of blank nanoparticles. Table S4: Physicochemical parameters of stigmasterol-loaded PLGA and PEG-PLA nanoparticles.
