# Supplementary material for: Evaluation of β-Sitosterol Loaded PLGA and PEG-PLA Nanoparticles for Effective Treatment of Breast Cancer: Preparation, Physicochemical Characterization, and Antitumor Activity
Source: Pharmaceutics. 2018 Nov 15;10(4):232. doi: 10.3390/pharmaceutics10040232 (PMC6321471; doi:10.3390/pharmaceutics10040232)
Supplement: Supplementary file 1 [file pharmaceutics-10-00232-s001.pdf]

pharmaceutics

Supplementary Materials: Evaluation of β-Sitosterol Loaded PLGA and PEG-PLA Nanoparticles for Effective Treatment of Breast Cancer: Preparation, Physicochemical Characterization, and Antitumor Activity

Moses Andima, Gabriella Costabile, Lorenz Isert, Albert J. Ndakala, Solomon Derese and Olivia M. Merkel

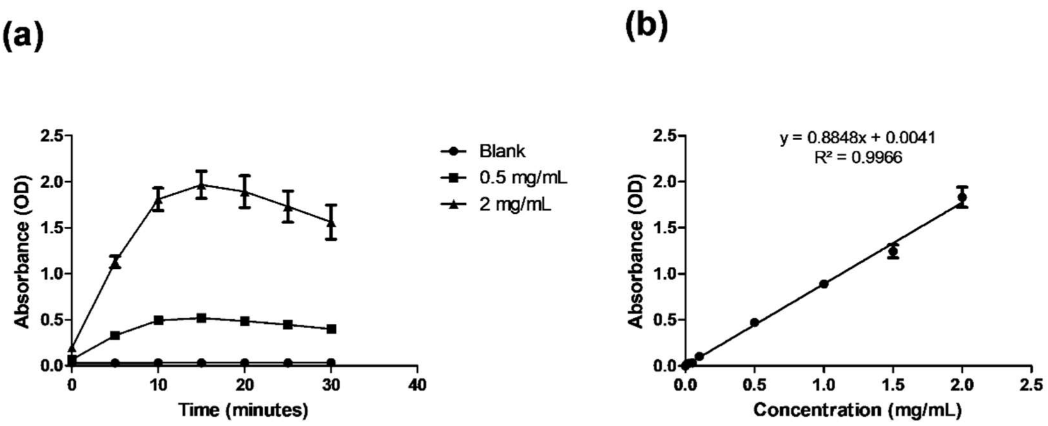

Figure S1. Time course for calorimetric reaction of β-sitosterol with Liebermann-Burchard reagent.

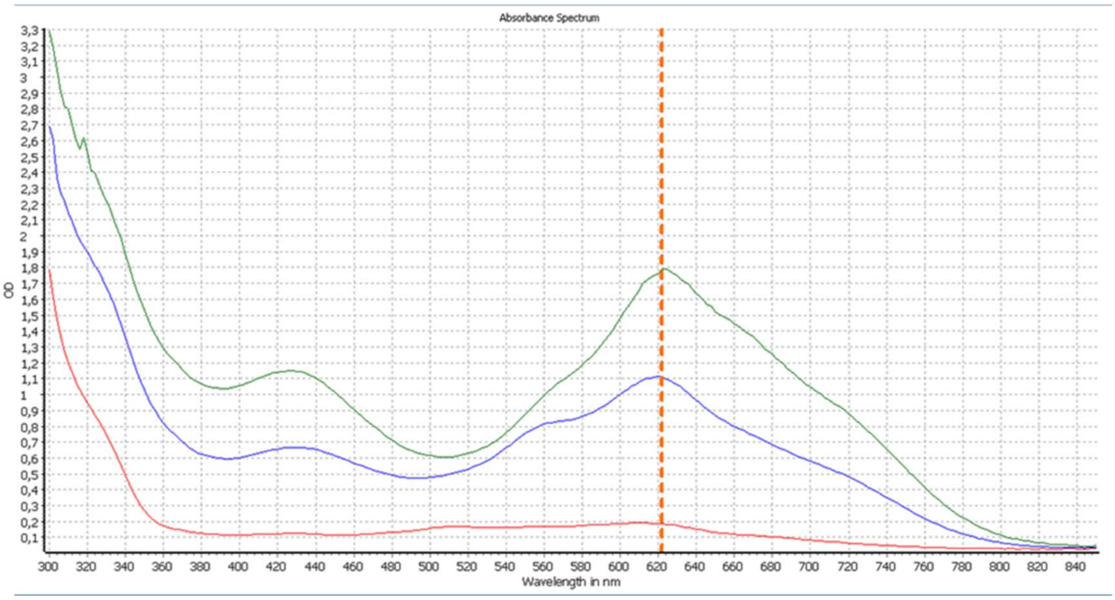

Figure S2. UV-Vis spectrum for calorimetric reaction of β-sitosterol with Liebermann-Burchard reagent.

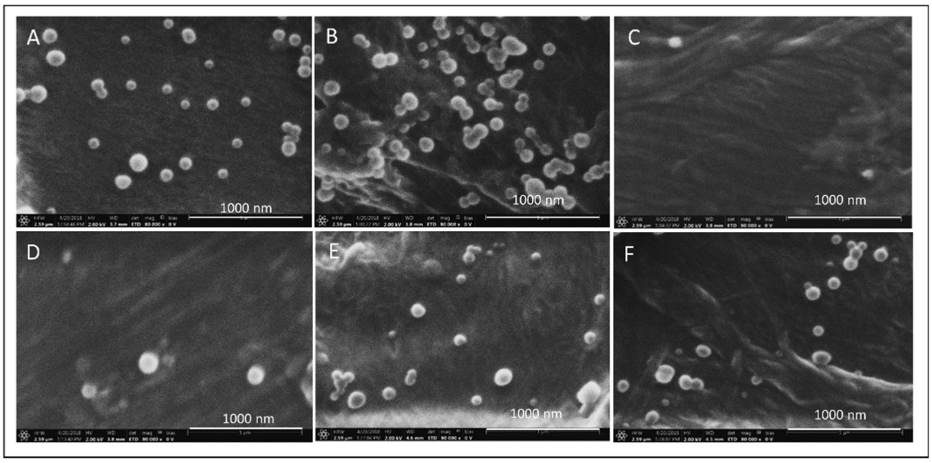

Figure S3. Surface morphology of nanoparticles after incubation in PBS and PBS/FBS.

Table S1 Method precision characterized by intra-assay and inter-assay precision.

	**Intra-Assay Precision**					**Inter-Assay Precision**				
**Concentration mg/mL**	**1**	**0.25**	**0.1**	**0.5**	**0.05**	**1**	**0.25**	**0.1**	**0.05**	**0.0125**
Absorbance for three runs (OD)	1.016	0.329	0.136	0.475	0.081	1.025	0.313	0.148	0.081	0.04
	1.001	0.335	0.139	0.493	0.082	1.028	0.304	0.146	0.082	0.038
	0.994	0.333	0.131	0.469	0.083	0.987	0.306	0.143	0.083	0.039
Mean Abs at 622 nm	1.004	0.332	0.135	0.479	0.082	1.013	0.308	0.146	0.082	0.039
St.Dev	0.011	0.003	0.004	0.012	0.001	0.023	0.005	0.003	0.001	0.001
% RSD	1.120	0.919	2.986	2.608	1.220	2.255	1.536	1.728	1.220	2.564

For intra-assay precision, samples were run in triplicate at five different concentration levels on the same day. For inter-assay precision samples were run at five different concentration levels on three different days, each in triplicate for each day. Precision is indicated by the %RSD with an acceptance of ± 2 %RSD.

Table S2. Spike recovery for determination of Liebermann-Burchard method for determination of β-Sit.

**Spiked Quantity (mg)**	**Recovered Quantity (mg)**	**Spike Recovery (%)**
0	0	0
0.4	0.3818	95.45
0.8	0.7767	97.0
1.5	1.554	103.60

Spike recovery was determined by standard addition method. Here, to 0.5 mL of stock nanoparticle suspension of β-Sit was added 1 mL of standard solution of β-Sit at three concentration levels; that is 0.4, 0.8 and 1.5 mg/mL. The spiked samples were extracted by shaking with DCM for 30 minutes. Absorbance of the recovered sample was measured using a microplate reader. Amount of β-Sit in the recovered sample was determined from the standard curve.

Recovery was determined using the equation below with acceptance level of ±5%

$$Recovery = \frac{[\beta - \text{Sit in spiked sample}] - [\beta - \text{Sit in unspiked sample}]}{[Spiked\ sample\ ]} x\ 100$$

Where [ ] = concentration.

Table S3 Physicochemical properties of blank nanoparticles.

Formulation Parameter	Sonication Amplitude (%)	Formulation Code	Physico-Chemical Properties		
			**Z-Average (nm)**	**PDI**	**Zeta Potential(mV)**
Effect of organic solvent	20	A1 (DCM)	335.7 ± 134.2	0.28 ± 0.02	−9.84 ± 6023
	25	A2 (DCM)	260.5 ± 62.81	0.072 ± 0.01	−10.3 ± 6.40
	30	A3 (DCM)	216.0 ± 96.83	0.138 ± 0.01	−10.6 ± 6084
	20	A4 (Acetone)	242.8 ± 55.87	0.004 ± 0.01	−13.7 ± 7.97
	25	A5 (Acetone)	221.9 ± 50.31	0.094 ± 0.02	−10.0 ± 5.54
	30	A6 (Acetone)	230.0 ± 75.53	0.129 ± 0.03	−10.3 ± 6.40
	20	A7 (Ethyl acetate)	182.0 ± 61.93	0.084 ± 0.01	−7.97 ± 5.05
	25	A8 (Ethyl acetate)	173.5 ± 60.97	0.094 ± 0.02	10.1 ± 5.54
	30	A9 (Ethyl acetate)	197.6 ± 69.28	0.129 ± 0.04	10.3 ± 6.40
Polymer concentration	20	B1 (5 mg/mL)	256.6 ± 103.9	0.183 ± 0.01	−14.3 ± 7.48
	25	B2 (5 mg/mL)	273.2 ± 90.52	0.254 ± 0.05	−12.7 ± 3.68
	30	B3 (5 mg/mL)	274.6 ± 121.0	0.143 ± 0.01	−13.7 ± 8.01
	20	B4 (10 mg/mL)	212.6 ± 13.71	0.146 ± 0.01	−10.55 ± 0.29
	25	B5 (10 mg/mL)	210.7 ± 5.43	0.116 ± 0.02	−10.75 ± 1.07
	30	B6 (10 mg/mL)	216.2 ± 5.40	0.156 ± 0.01	−9.33 ± 0.50
	20	B7 (15 mg/mL)	211.4 ± 13.27	0.148 ± 0.01	−11.27 ± 0.35
	25	B8 (15 mg/mL)	205.9 ± 4.81	0.135 ± 0.01	−10.82 ± 0.33
	30	B9 (15 mg/mL)	206.4 ± 9.29	0.175 ± 0.02	−10.19 ± 0.11
	20	B10 (20 mg/mL)	194.2 ± 0.21	0.044 ± 0.02	−13.15 ± 3.18
	25	B11 (20 mg/mL)	202.1 ± 9.83	0.118 ± 0.01	14.00 ± 1.13
	30	B12 (20 mg/mL)	184.7 ± 0.57	0.067 ± 0.01	14.45 ± 1.48
PVA concentration (% *w/v*)	20	C1 (1 %, *w/v*)	219.6 ± 16.54	0.06 ± 0.01	−14.6 ± 0.49
	20	C2 (2 % *w/v*)	175.5 ± 46.84	0.042 ± 0.01	−15.0 ± 6.38
Aqueous/Org phase ratio	20	D1 (2:1, vol:vol)	321.0 ± 69.85	0.093 ± 0.04	−8.31 ± 6.81
	20	D2 (5:1, vol:vol)	219.5 ± 51.70	0.064 ± 0.01	−14.3 ± 7.98
Duration of solvent evaporation (Hours)	20	E1 (4 hours)	233.5 ± 3.32	0.045 ± 0.01	−12.8 ± 6.42
	25	E2 (4 hours)	217.8 ± 0.99	0.107 ± 0.01	−14.3 ± 7.48
	30	E3 (4 hours)	207.2 ± 5.37	0.135 ± 0.02	−13.7 ± 8.01
	20	E4 (15 hours)	178.8 ± 4.60	0.137 ± 0.01	−11.5 ± 5.00
	25	E5 (15 hours)	175.2 ± 2.33	0.042 ± 0.01	−12.7 ± 3.68
	30	E6 (15 hours)	178.1 ± 2.12	0.127 ± 0.01	−12.5 ± 3.11

The effect of different nanoparticle formulation parameters were investigated one at a time by keeping other factors constant and varying one factor along with sonication amplitude. Results are presented as Mean ± SD, n = 3.

Table S4. Physicochemical parameters of stigmasterol-loaded PLGA and PEG-PLA nanoparticles.

Polymer Type	Feeding Drug Concentration (mg/mL)	Physicochemical Parameters				
		**Particle size (nm)**	**PDI**	**Zeta Potential (mV)**	**Loading Efficiency (%)**	
					**Method A**	**Method B**
PLGA	1	200.1 ± 87.20	0.134 ± 0.01	−8.61 ± 5.04	34.05 ± 0.44	83.17 ± 1.17
	2	206.0 ± 68.70	0.111 ± 0.02	−8.17 ± 6.27	51.95 ± 8.70	77.38 ± 3.68
	4	218.7 ± 83.40	0.114 ± 0.02	−8.98 ± 4.72	72.21 ± 3.83	95.95 ± 0.01
	8	231.8 ± 89.51	0.107 ± 0.02	−8.39 ± 4.96	ND	ND
PEG-PLA (R25)	1	240.3 ± 102.2	0.182 ± 0.01	−24.6 ± 4.48	64.84 ± 1.46	81.91 ± 2.94
	2	245.3 ± 84.85	0.09 ± 0.01	−19.6 ± 6.48	51.74 ± 0.07	83.99 ±
	4	245.0 ± 77.63	0.075 ± 0.01	−23.7 ± 9.48	57.90 ± 0.024	82.07 ± 4.95
	8	266.2 ± 72.89	0.058 ± 0.01	−20.6 ± 8.79	ND	ND

Effect of varying feeding drug concentration on particle size, size distribution, zeta potential and drug loading efficiency were investigated. Concentration of polymer used was fixed at 20 mg/mL, Ethyl acetate was used for PLGA nanoparticle formulation while acetone was used for PEG–PLA nanoparticle formulations. Sonication power amplitude was 30 %, organic to aqueous phase ratio being 5:1, duration of solvent evaporation 15 hours.
